# The recombinant IL-35 and anti-Ebi3 antibody administration before implantation modulate immune regulation and fetal outcomes in an abortion-prone mouse model

**DOI:** 10.3389/fimmu.2025.1648641

**Published:** 2025-11-19

**Authors:** Anna Slawek, Paulina Kubik, Mateusz Psurski, Anna Ewa Kedzierska, Anna Chelmonska-Soyta

**Affiliations:** 1 Department of Experimental Therapy, Hirszfeld Institute of Immunology and Experimental Therapy, Polish Academy of Sciences, Wroclaw, Poland; 2 Department of Immunology, Pathophysiology and Veterinary Preventive Medicine, Wroclaw University of Environmental and Life Sciences, Wroclaw, Poland

**Keywords:** IL-35, Bregs, Tregs, abortion-prone, fetal blood flow, B10, T2-MZP, iTr35 cells

## Abstract

**Introduction:**

Interleukin-35 (IL-35), consisting of two subunits - Ebi3 and p35, is a pleiotropic anti-inflammatory cytokine implicated in fetal tolerance and pregnancy maintenance. Reduced IL-35 levels in abortion-prone mice and women with recurrent miscarriage suggest its deficiency contributes to pregnancy failure. In abortion-prone mice, IL-35 administration during mid-term gestation rescued pregnancy. However, it is unclear whether IL-35 administration before implantation (during the time of the first recognition of paternal antigens) can expand regulatory lymphocyte pools and restore maternal tolerance. Therefore, this study aimed to investigate the influence of intraperitoneal administration of recombinant IL-35 (rIL-35) and anti-Ebi3 antibody shortly after mating on successful pregnancy, fetal blood flow, and the profiles of several types of regulatory cells in a murine abortion-prone model.

**Methods:**

rIL-35 and anti-Ebi3 antibody were administered on 0 days *post coitum* (dpc). The embryos were imaged in PW Doppler mode on the 14th day of pregnancy. The frequencies of different subpopulations of Bregs (B10, MZ, T2-MZP, FO), Tregs, iTr35, γδ T, Th17 and NK cells were measured in uterine-draining lymph nodes, spleens and decidua using flow cytometry. The concentrations of Th1/Th2/Th17 cytokines in the serum were analyzed.

**Results:**

The main finding of our study is that we did not observe any differences in abortion rates between the groups. In the group that received the neutralizing antibody, a lower embryonic heart rate, lower circulatory competence of the fetal placenta, and elevated serum Th17/Th2 cytokine concentrations were observed. IL-35 administration increased the frequency of B10 and IL-35-producing B10 and regulatory T cells at the periphery and NK^IL-35+^ and CD19^+^IL-35^+^ but not Treg cells in the decidua.

**Conclusions:**

A single administration of IL-35 shortly after mating, does not have an anti-abortion effect. It exhibits a multifaceted effect on immune regulatory cells and IL-35 neutralization results in decreased embryonic heart rate and impaired placental–fetal circulation.

## 1 Introduction

Coordinated interactions between the maternal immune and reproductive systems are crucial for a pregnancy. The cytokine repertoire and their concentrations regulate the systemic and local pro-/anti-inflammatory milieu, which is critical for pregnancy maintenance. Successful pregnancies depended on timely and efficient transitions from pro- to anti-inflammatory stages. The number and phenotype of cytokine-producing lymphocytes change during the course of normal pregnancy, adapting to the type and strength of paternal antigen expression. In pathological pregnancy, their repertoire is disturbed, leading to cytokine imbalance (1). In 1993, Wegmann (2, 3) proposed a hypothesis known as the “T helper 2 (Th2) phenomenon” which consisted of skewing the Th1/Th2 balance toward the Th2 side. Next, this paradigm was expanded to the Th1/Th2/Th17 and regulatory T (Treg) cell paradigms (4), in which Th2 and Treg cells are responsible for maternal tolerance toward fetal alloantigens, while Th1 and Th17 cells are responsible for spontaneous abortion. More recent studies have highlighted inducible regulatory T cells producing IL-35 (iTr35), a distinct subset of regulatory T cells defined by their stable phenotype and potent immunosuppressive capacity through secretion of interleukin-35 (IL-35) (5). In addition to T cells, regulatory B lymphocytes, classically associated with IL-10 production can also synthesize IL-35 - a heterodimer composed of two subunits, Ebi3 and p35 (6). While Tregs and iTr35 cells are the main sources of IL-35, other immune cells (e.g., Bregs, tolerogenic dendritic cells) (7–9), tumor-associated cells (monocytes/macrophages and Treg lymphocytes (10, 11), and non-immune cells (e.g., tumor, muscle, endothelial, trophoblast) (12–16) also produce this cytokine. IL-35 has been implicated in both pathological and physiological conditions, including cancer and pregnancy. IL-35 and its subunits p35 and Ebi3 are differentially expressed in abortion-prone (AP; crossbreeding of CBA/J♀ × DBA/2J♂) and normal (NP; crossbreeding of CBA/J♀ × Balb/c♂) mice placentas. Reduced IL-35 expression in AP females suggests an important role in local pregnancy regulation (15). In the same model, CD19^+^IL35^+^ lymphocyte frequency decreased in the uterus and decidua on days 3 and 14 of pregnancy, respectively (6). Importantly, this cytokine is responsible for propagating tolerance by ‘infecting’ other lymphocytes and downregulating unfavorable Th17 response in mouse pregnancy (17–22). Similarly, lower IL-35 serum levels have been reported in women with recurrent spontaneous abortion compared with women experiencing normal early pregnancy (23). Together, these findings support the idea that IL-35 is essential for pregnancy maintenance and may represent a potential therapeutic target in pregnancy complications. In human therapeutic approaches to restore cytokine balance during pregnancy and miscarriage prevention include blocking pro-inflammatory cytokines (e.g., with TNF-α antagonists), administration of anti-inflammatory cytokines or using immunomodulatory agents (e.g., progesterone, glucocorticoids, antibiotics), and intravenous immunoglobulin infusion (24, 25). However, their effects remain inconsistent (26). In contrast, in mice models experimental studies have demonstrated that exogenous administration of anti-inflammatory cytokines (IL-10, IL-3, GM-CSF, IL-35) during mid-gestation reduces fetal resorption and enhances tolerance, primarily by increasing Th2 cytokines and Treg frequency (15, 27–29). On the other hand, studies by Ann Zenclussen’s group have shown that an increased number of Treg and Breg lymphocytes, achieved through adoptive transfer, is required for successful pregnancy in the abortion-prone mouse model specifically before implantation (30). According to current knowledge, Treg-produced IL-35 affects B lymphocytes by differentiating them into IL-35–producing Bregs, while IL-35–producing Bregs can reciprocally act on Tregs (22). However, it remains unclear whether IL-35 administered during the pre-implantation period can modulate these mutual interactions and expand the pool of IL-35–producing lymphocytes. We hypothesized that administration of IL-35 during the pre-implantation period would promote pregnancy success in the abortion-prone mouse model by expanding regulatory B and T lymphocyte populations and restoring immune tolerance. To test this, we analyzed B cell subsets (B10, marginal zone, transitional-2 MZP, follicular B cells), regulatory and effector T cell populations (Tregs, iTr35, γδ T cells, Th17 cells), and NK cells. Additionally, given the pleiotropic effects of IL-35 on non-immune cells such as endothelium, we assessed fetal blood flow in mid-gestation. Finally, we examined the distribution, frequency, and intracellular cytokine expression (IL-35, IL-10, TGF-β, IL-17) of pregnancy-related regulatory lymphocytes in both peripheral and local immune compartments.

## 2 Materials and methods

### 2.1 Animals

This study was conducted in accordance with the recommendations of the 1^st^ Local Ethics Committee for Experiments on Animals at the Institute of Immunology and Experimental Therapy, Wroclaw No 052/2023/P1. Adult (8-week-old) female CBA/J and male DBA/2J mice were purchased from Charles River Laboratory (France) and housed at 12:12 h constant light-to-dark ratio under specific pathogen-free (SPF) conditions. The estrous cycle was controlled by cytocolor staining (Merck Millipore, Germany) according to the manufacturer’s instructions. During the proestrus phase, the females were mated with DBA/2J males. Mating was confirmed by the presence of a vaginal plug and defined as 0 day *post coitum* (dpc). Pregnancy was confirmed by ultrasound measurements using the Vevo 2100 system (Visualsonics, Amsterdam, Netherlands) and observing the number of fetuses or implantation sites on day 14 of pregnancy (the period of pregnancy when the placenta is fully developed and feto-maternal contact is stabilized). The AP pregnancy (CBA/J♀×DBA/2J♂) mice were categorized into three groups and investigated: mice after intraperitoneal rIL35 (Merck Life Science; 2 µg), anti-EBI3/IL35 neutralizing antibody (Merck Life Science, clone: V1.4C4.22; 5 µg) or phosphate-buffered saline (PBS) administration on 0 dpc (n=7 per group). The dose of rIL-35 was established after careful analysis of published studies using mouse models in which rIL-35 was administered intraperitoneally (31–34). We considered only those studies that applied a single-dose treatment. We based our experiment on the results of Zheng et al. (34) who tested IL-35 at 1, 10, and 100 µg/kg body weight (corresponding to approximately 0.02, 0.2, and 2 µg for a 20 g mouse) and demonstrated dose-dependent suppression of D-GalN/LPS-induced liver injury, as assessed by histology (HE staining) and serum ALT activity. In that study, the 100 µg/kg dose produced the most pronounced effect on cytokine production. Based on these findings, we considered 2 µg/mouse an appropriate and effective dose for our experiments. In contrast, only one publication reported the use of an anti-IL-35 antibody in mice (35). In this study, a single dose of 5 µg/mouse was administered. Accordingly, we adopted the same dose in our experiments. Finally, we also took into account results from experiments with IL-10, a tolerogenic cytokine with functions similar to IL-35. IL-10 was administered at higher doses (2.5–4 µg/mouse) and was shown to be safe for females while reducing the number of resorbed fetuses in the LPS-treated µMT mouse model (36, 37). These comparative data further supported the safety and rationale of our chosen IL-35 dosing strategy. On day 14 of pregnancy, after bleeding from the jugular vein, the females were euthanized by cervical vertebral dislocation and the spleen, decidua, and uterine-draining lymph nodes were dissected. We also assessed the number of normal fetuses and resorbed embryos on day 14 of pregnancy. The abortion rate was calculated as the ratio of resorbed fetuses to the total number of implantation sites. All mice were kept under the same conditions with high sanitary and hygienic standards.

### 2.2 Blood flow analyses in umbilical cord

On gestational day 14, the mice were anesthetized. Anesthesia was induced with 2.5–3% isoflurane and subsequently maintained at 1.75–2% for the duration of the procedure. The isoflurane was delivered in synthetic air (80% N_2_, 20% O_2_) at a flow rate of 0.6-0.8 L/min. Following induction, animals were allowed to stabilize for 3–5 minutes before imaging to ensure hemodynamic stability. The physiological status of each mouse was monitored throughout the procedure via ECG and respiration rate. The abdominal fur was shaved and the animals were positioned on a heated ultrasound stage. All viable embryos in each dam (from 2 to 8) were imaged in PW Doppler mode using a VisualSonics Vevo 2100 ultrasound system equipped with an MS550S transducer. Imaging was performed in a plane that allowed for the clearest visualization of the umbilical vessels. PW Doppler sequences were recorded with the following standardized settings: transmission frequency (Tx freq) 32 MHz, pulse repetition frequency (PRF) 10 kHz, Doppler gain 30 dB, wall filter 150 Hz, and gate size 0.31 mm. To ensure accuracy, the Doppler angle was manually adjusted for each measurement to align with the direction of blood flow within the vessel (angle correction), and the beam angle was maintained at 0 degrees. To maintain a consistent workflow and minimize total anesthesia time, embryos were typically imaged sequentially based on their position within the uterine horns. Upon completing the imaging procedure, the animals received buprenorphine (0.1 mg/kg body weight). This step was included to adhere to Polish animal welfare regulations, which mandate the administration of analgesia prior to potentially painful procedures such as blood collection, even when followed by euthanasia. After 10 min, blood was collected from the ophthalmic venous sinus and next euthanasia by cervical dislocation was performed, followed by organs collection for further analysis. A comprehensive panel of parameters, including peak velocity (PV), mean velocity (MV), pulsatility index (PI), resistive index (RI), velocity time integral (VTI), and fetal heart rate (HR) were analyzed from the PW Doppler images using VevoLab 5.10.0 software. This comprehensive set was chosen to allow for a distinct assessment of fetal cardiac function (e.g., HR, PV) and placental vascular competence (e.g., PI, RI), providing a holistic view of fetal-placental hemodynamics.

### 2.3 Splenocyte and lymph node cell isolation

Freshly dissected spleens were weighed and immediately pushed through a 40-μm sieve to obtain a single-cell suspension in 0.84% ammonium chloride (Merck Life Science) solution (Inaba et al., 1995). After red blood cell lysis (1 min. on ice), the suspension was centrifuged (300× *g*, 10 min) and washed twice in phosphate-buffered saline (PBS, Merck Life Science). Para-aortic lymph nodes were pushed through a 40-μm sieve into PBS and subsequently centrifuged (300× *g*, 10 min). The cells were counted using a Bürker chamber hemocytometer.

### 2.4 Isolation of cells from mouse decidua

After euthanizing the pregnant mice (14 dpc), the placentae were removed from the uteri, stripped of all membranes, and rinsed with cold Hank’s Balanced Salt Solution (HBSS) (Biowest) containing 10% FBS (Biowest). The maternal surface of the placenta (decidua) was separated from the fetal surface as described by Croy (38). Decidual tissues from all placentae of morphologically normal fetuses were pooled, pushed through a 40-μm sieve in PBS, and centrifuged (300× *g*, 10 min). The cells were counted using a Bürker chamber hemocytometer.

### 2.5 Flow cytometry

The cells (1×10^6^) were stained with appropriate antibodies. For intracellular staining, the cells obtained from the spleen, para-aortic lymph nodes and decidua were incubated in RPMI-1640 medium (Biowest) (supplemented with 10% FBS (Biowest), L-glutamine (Biowest, diluted 1:100), penicillin/streptomycin stock (Biowest, diluted 1:100)) with 1 μL Leukocyte Activation Cocktail (LAC; 0.1 μg/mL phorbol 12-myristate 13-acetate (PMA, Cayman) + 1 μg/mL ionomycin (Cayman)), 10 μg/mL Brefeldin A (BioLegend), and 2 μM Monensin A (BioLegend) in a tissue culture incubator at 37 °C, 5% CO_2_ for 4 h. Before surface staining, Zombie UV™ Fixable Viability Kit (BioLegend) and TruStain FcX™ (anti-mouse CD16/32, BioLegend) antibody were used. Surface staining were performed for 20 min at 4 °C with an appropriate cocktail of fluorescently labeled antibodies (for regulatory T and B cells - anti-mouse B220 (BioLegend, clone: RA3-6B2), CD23 (BioLegend, clone: B3B4), CD21/35 (BioLegend, clone: 7E9), CD24 (BioLegend, clone: M1/69), IgM (BioLegend, clone: RMM-1), CD19 (BioLegend, clone: 6D5), CD5 (BioLegend, clone: 53-7.3), CD1d (BioLegend, clone: 1B1), CD138 (BioLegend, clone: 281-2), CD3 (BioLegend, clone: 17A2), CD4 (BioLegend, clone: RM4-4), CD25 (BioLegend, clone: 3C7), Tγδ (BioLegend, clone: GL3) – in spleen and para-aortic lymph nodes). Additionally, splenic NK cells (anti-mouse NKp46, BioLegend, clone 29A1.4), CD3 (BioLegend, clone 17A2), CD11b (BioLegend, clone M1/70), CD27 (BioLegend, clone LG.3A10), and CD49b (BioLegend, clone DX5) were labeled. In decidua, anti-mouse CD3 (BioLegend, clone: 17A2), CD4 (BioLegend, clone: RM4-4), CD19 (BioLegend, clone: 6D5), NKp46 (BioLegend, clone: 29A1.4), CD49a (BioLegend, clone: HMα1), CD49b (BioLegend, clone: DX5), Tγδ (BioLegend, clone: GL3), CD45 (BioLegend, clone: 30-F11) antibodies were used. After incubation, the cells were washed twice and intracellular staining was performed according to the manufacturer’s protocol (True-Nuclear Transcription Factor Buffer Set, (BioLegend). Briefly, the cells were fixed for 30 min at 4 °C in dark, washed with FACS buffer (PBS, 0,5 mM EDTA (Merck Life Science), 0,002% sodium azide (Merck Life Science), 1% FBS) and left at 4 °C in dark overnight. The cells were then washed with Permeabilization Buffer. Before extracellular staining, TruStain FcX™ (anti-mouse CD16/32) antibody was used. The cells were stained for 1 h at 4 °C in dark with anti-mouse IL-10 (BioLegend, clone: JES5-16E3), Ebi3 (R&DSystems, clone: 355022), p35 (R&DSystems, clone: 27537), Helios (BioLegend, clone: 22F6), Nrp1 (BioLegend, clone: 3E12), FOXP3 (BioLegend, clone: MF-14), ROR gamma t (R&DSystems, clone: 1181A), IL-17A (BioLegend, clone: TC11-18H10.1) antibodies or appropriate isotype controls in the same concentration as specific antibodies. The cells were washed twice with the Permeabilization Buffer (BioLegend) and immediately processed using an LSR Fortessa cell analyzer (BD Biosciences). All analyses were performed using the FlowJo software (BD Biosciences). For t-SNE analysis populations gated on CD19^+^ cells were downsampled to 50,000 events per sample and concatenated into 660,000 events using FlowJo v10.10.0, ensuring an equal contribution from each donor. The merged dataset was then analyzed using the t-distributed Stochastic Neighbor Embedding (t-SNE) tool in FlowJo v10.10.0 to project the flow cytometry data into a lower-dimensional space for visualization (39). FlowSOM clustering was conducted alongside t-SNE to provide clustering outputs that could be visualized in these reduced dimensions. Twelve meta-clusters were established to identify the main subpopulations. The Cluster Explorer function was used to explore phenotypic similarities and differences. Quantitative analyses of rare subpopulations were performed on the full datasets, and thus were not affected by bias introduced by downsampling.

### 2.6 ELISA

Mouse serum (frozen at −80 °C) was assayed using a commercially available ELISA kit for IL-35 and TGF-beta (Biolegend) according to the manufacturer’s protocol. Moreover, the ProcartaPlex™ Mouse Combinable Panels have been used for detection of multiple analytes (GM-CSF, IFN gamma, IL-1 beta, IL-2, IL-4, IL-5, IL-6, IL-9, IL-10, IL-12p70, IL-13, IL-17A (CTLA-8), IL-18, IL-22, IL-23, IL-27, IL-33, TNF alpha) from mouse serum (ThermoFisher Scientific).

### 2.7 Statistical analysis

All statistical analyses were performed using GraphPad Prism 8.0.2. The shape of the data distribution was assessed using the Shapiro–Wilk or Kolmogorov–Smirnov normality test and analysis of quantile-normal plots. We used the Kruskal–Wallis test (nonparametric) with Dunn’s multiple comparison *post-hoc* test or one-way analysis of variance (parametric) with Tukey’s multiple comparison test to compare the data from the three tested groups, or Dunnett’s multiple comparison test to CTRL. *p* < 0.05 was considered statistically significant. All ultrasound statistical analyses treated the dam as the experimental unit to address the non-independence of littermates. The specific statistical test was selected for each parameter based on its data distribution, assessed using the Shapiro-Wilk test. For normally distributed data, a linear mixed-effects model (LMM) was fitted, specifying ‘treatment’ as a fixed effect and ‘dam’ as a random effect. Significant main effects were followed by Dunnett’s multiple comparisons test. For non-normally distributed data, median values were calculated for each dam and compared among groups using the Kruskal-Wallis test, with Dunn’s test used for *post-hoc* comparisons. A *p-value<0.05* was considered statistically significant for all analyses. In our analysis, each biological endpoint (e.g., cytokine level, lymphocyte subset, ultrasound parameter) was treated as a distinct hypothesis derived from independent biological rationales. Comparisons were therefore performed separately across the three study groups for each endpoint and tests were applied as described above. These procedures are widely accepted in biomedical research as adequate correction methods within a family of related comparisons (40, 41). We did not apply a global false discovery rate correction across all heterogeneous endpoints, as these represent biologically independent hypotheses. Pooling them into a single correction procedure could introduce over-adjustment and increase the risk of false negatives, a concern highlighted in the statistical literature (42, 43).

## 3 Results

### 3.1 The effect of IL-35 on pregnancy success and circulatory parameters of 14^th^-day fetuses

**Figure 1:**
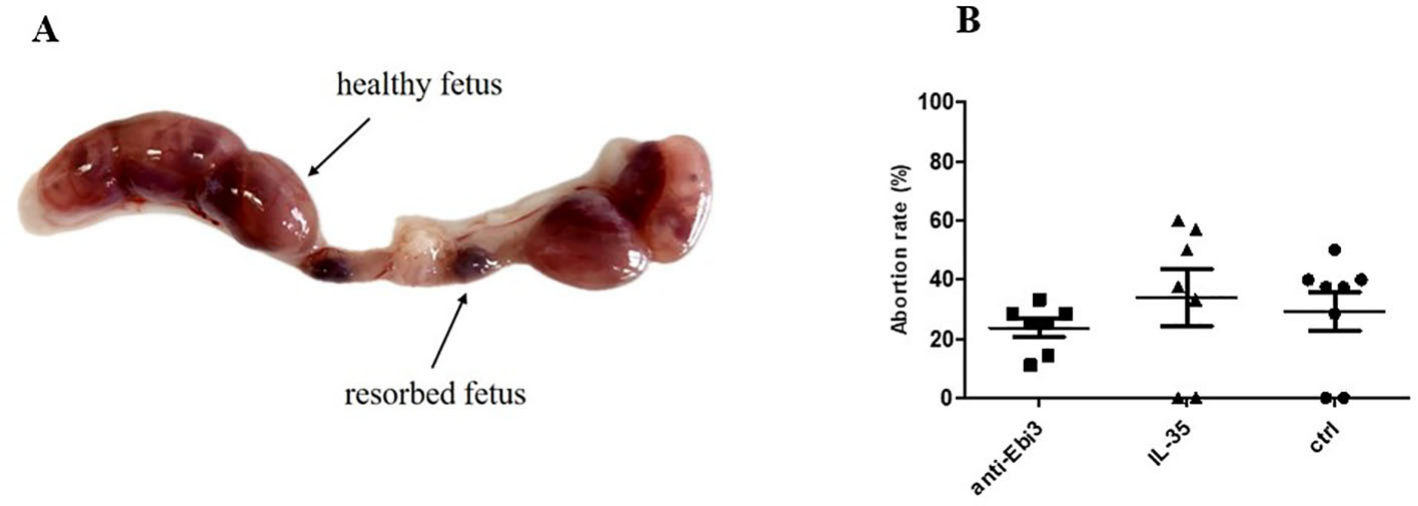
Representative picture of uterus from abortion-prone mice (CBA/J♀×DBA/2J♂) **(A)** and abortion rate **(B)**; n=7. Data are expressed as mean ± standard error of the mean (SEM). Normality was assessed using the Shapiro–Wilk normality test. No differences between examined groups as analyzed by the Kruskal–Wallis test.

**Figure 2:**
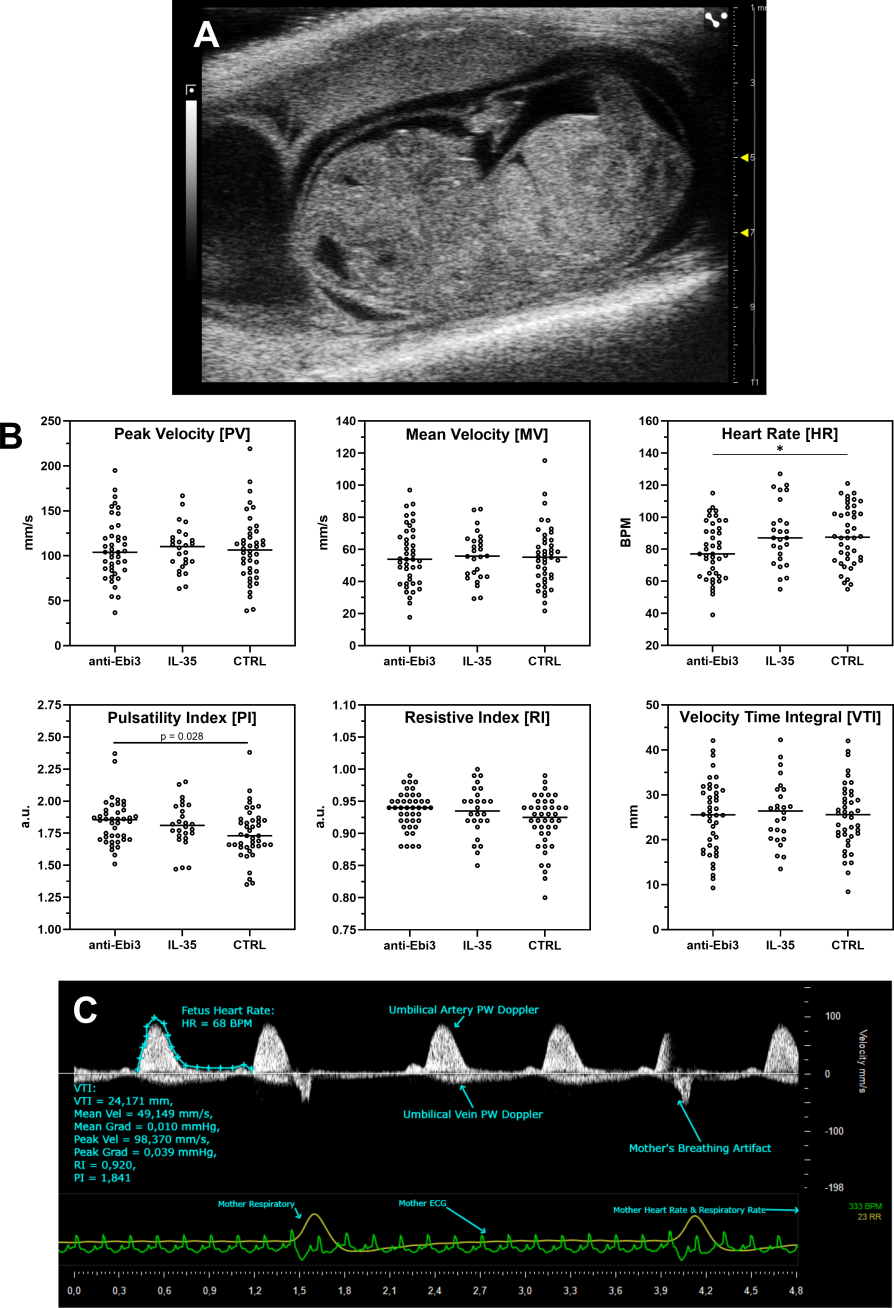
Ultrasound analysis of umbilical cord blood flow following treatment. **(A)** Representative sagittal view of a murine fetus. **(B)** Quantification of key Doppler parameters. Each dot represents a measurement from an individual fetus, plotted to show the full data distribution. Crucially, all statistical analyses were performed using the dam as the experimental unit to account for the litter effect. Normally distributed parameters (Peak Velocity, Mean Velocity, Velocity Time Integral, Heart Rate) were analyzed with a linear mixed-effects model, while non-normally distributed parameters (Pulsatility Index, Resistive Index) were analyzed with a Kruskal-Wallis test on dam-level medians. Cetral line in each dataset rep[resents median; *p<0.05 indicates a significant difference from the control group, determined by Dunnett’s or Dunn’s *post-hoc* test, respectively. **(C)** Representative Pulse Wave (PW) Doppler image showing key measured parameters.

**Figure 1A** shows a representative picture of the uterus from abortion-prone mice with healthy and resorbed fetuses. Although in each examined group of animals we observed resorbed fetuses, we did not observe any differences in abortion rate between the examined groups (**Figure 1B**) after single dose administration of both rIL-35 and antibody anti-Ebi3. However, in the group that received the blocking antibody, ultrasound examination showed a significantly lower embryonic HR (**Figure 2B**) and lower circulatory competence of the fetal placenta, as indicated by an increased PI (**Figure 2B**) compared to the control group. Other parameters of ultrasound examination remained unchanged. IL-35 administration did not affect any of the parameters examined by ultrasound. **Figure 2A** shows a representative image of a murine fetus in the sagittal plane.

### 3.2 IL-35 administration increases the B10 and IL-35-producing B10 cells proportion

**Figure 3:**
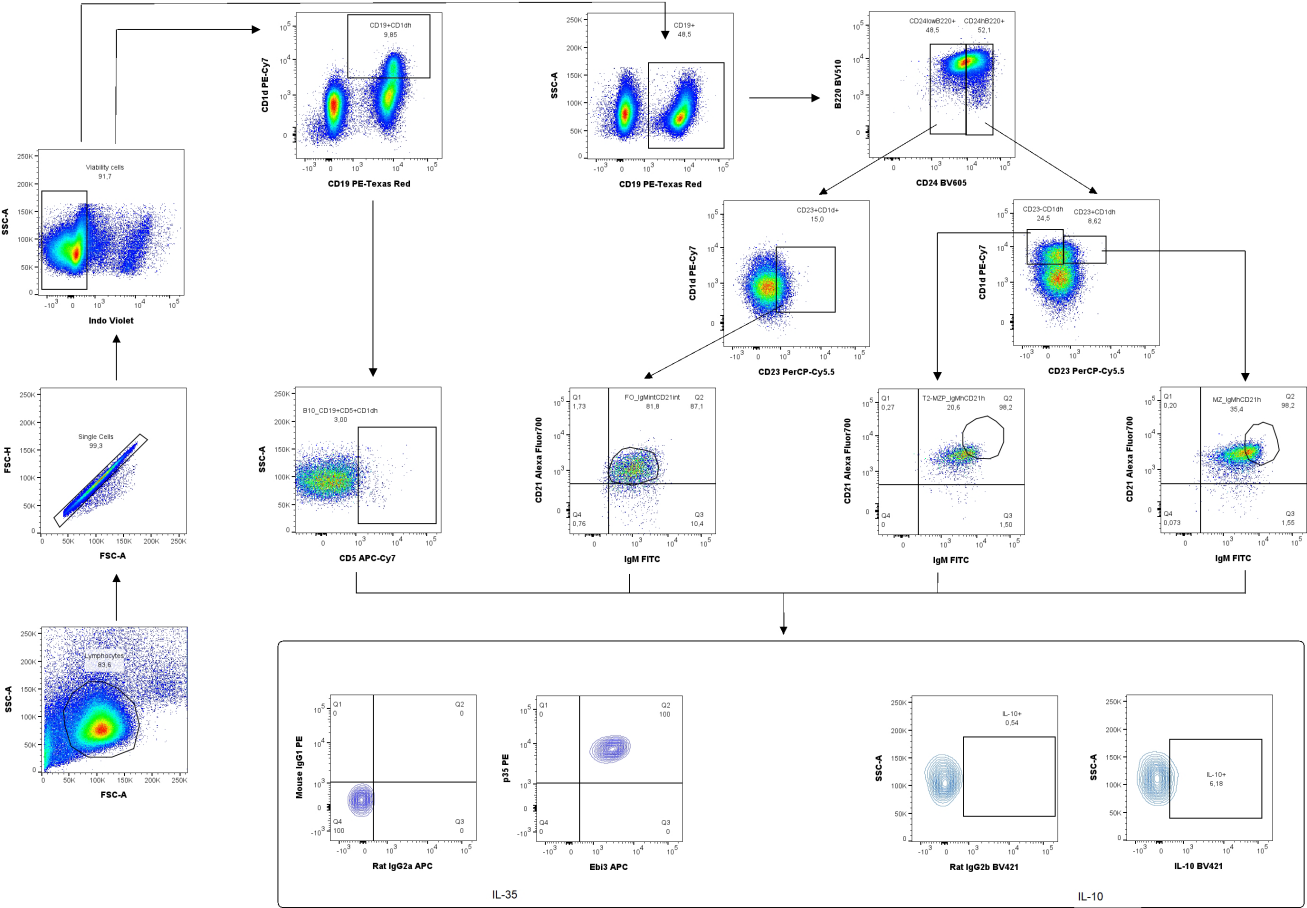
Representative dot-plots showing the gating strategy of regulatory B cells: B10 (CD19^+^CD5^+^CD1d^hi^), marginal zone (MZ-CD19^+^CD24^hi^B220^+^CD23^−^CD1d^hi^IgM^hi^CD21^hi^), transitional-2 marginal zone precursor (T2-MZP-CD19^+^CD24^hi^B220^+^CD23^+^CD1d^hi^IgM^hi^CD21^hi^), follicular cells (FO -, CD19^+^CD24^low^B220^+^CD23^+^CD1d^+^IgM^int^CD21^int^) producing/expressing interleukin (IL)-35 and IL-10. For cytokine analysis, we used appropriate isotype controls in the same concentration as specific antibodies. For surface antigens FMO controls were used.

**Figure 4:**
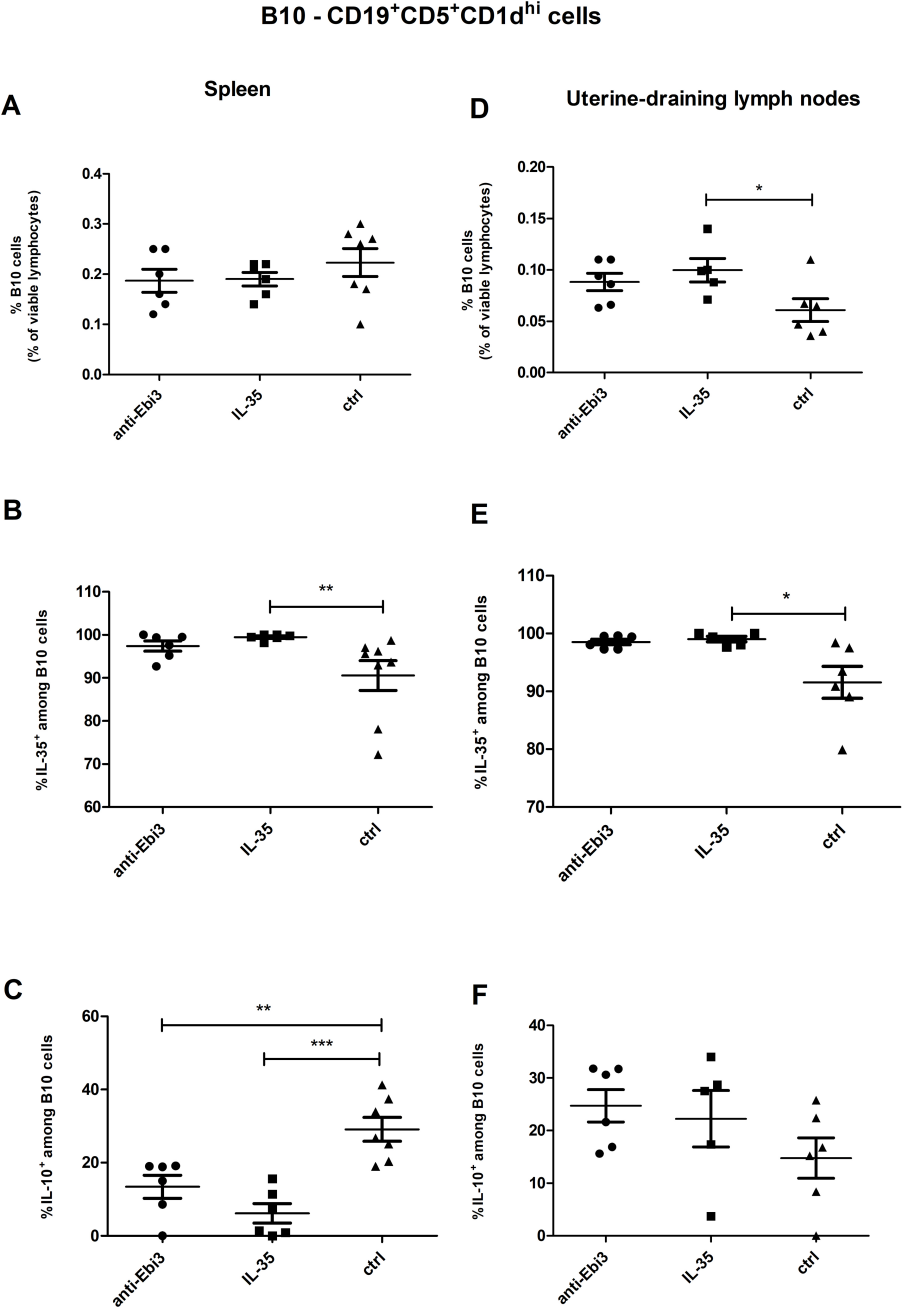
The percentage of B10 cells within viable lymphocytes in spleen **(A)** and uterine-draining lymph nodes **(D)**. Frequencies of B10 cells with **(B)** interleukin (IL)-35 and **(C)** IL-10 expression within B10 cells in the spleen of DBA/2J-mated CBA/J females at 14 days *post coitum* (dpc), analogously for uterine-draining lymph nodes **(E, F)** n=7. Data are expressed as mean ± standard error of the mean (SEM). Normality was assessed using the Shapiro–Wilk normality or Kolmogorov–Smirnov tests. *p < 0.05, **p < 0.01, ***p < 0.001 as analyzed by the one-way analysis of variance with Tukey’s multiple comparison test or Krauskal–Wallis test with Dunn’s multiple comparison test.

According to the gating strategy depicted in **Figure 3**, we were able to identify the following phenotypes of Breg cells: B10 cells (CD19^+^CD5^+^CD1d^hi^), marginal zone cells (MZ-CD19^+^CD24^hi^B220^+^CD23^−^CD1d^hi^IgM^hi^CD21^hi^), transitional-2 marginal zone precursor cells (T2-MZP-CD19^+^CD24^hi^B220^+^CD23^+^CD1d^hi^IgM^hi^CD21^hi^), and follicular cells (FO-CD19^+^CD24^low^B220^+^CD23^+^CD1d^+^IgM^int^CD21^int^). The frequency of B10 cells in the uterine-draining lymph nodes but not in the spleen of IL-35 group increased compared to the control group (**Figures 4D, A**, respectively). Moreover, the frequency of these cells with IL-35 expression was higher in the IL-35 group both in the spleen and in the lymph nodes (**Figures 4B, E**). The opposite was true for IL-10-producing B10 cells. After administering IL-35 and anti-Ebi3 antibody, the frequency of B10^IL-10+^ cells decreased in the spleen (**Figure 4C**). The proportion of B10^IL-10+^ cells did not change in uterine-draining lymph nodes between examined groups (**Figure 4F**).

### 3.3 Neutralizing anti-Ebi3/IL-35 antibody administration increased T2-MZP cell frequency in the spleen

**Figure 5:**
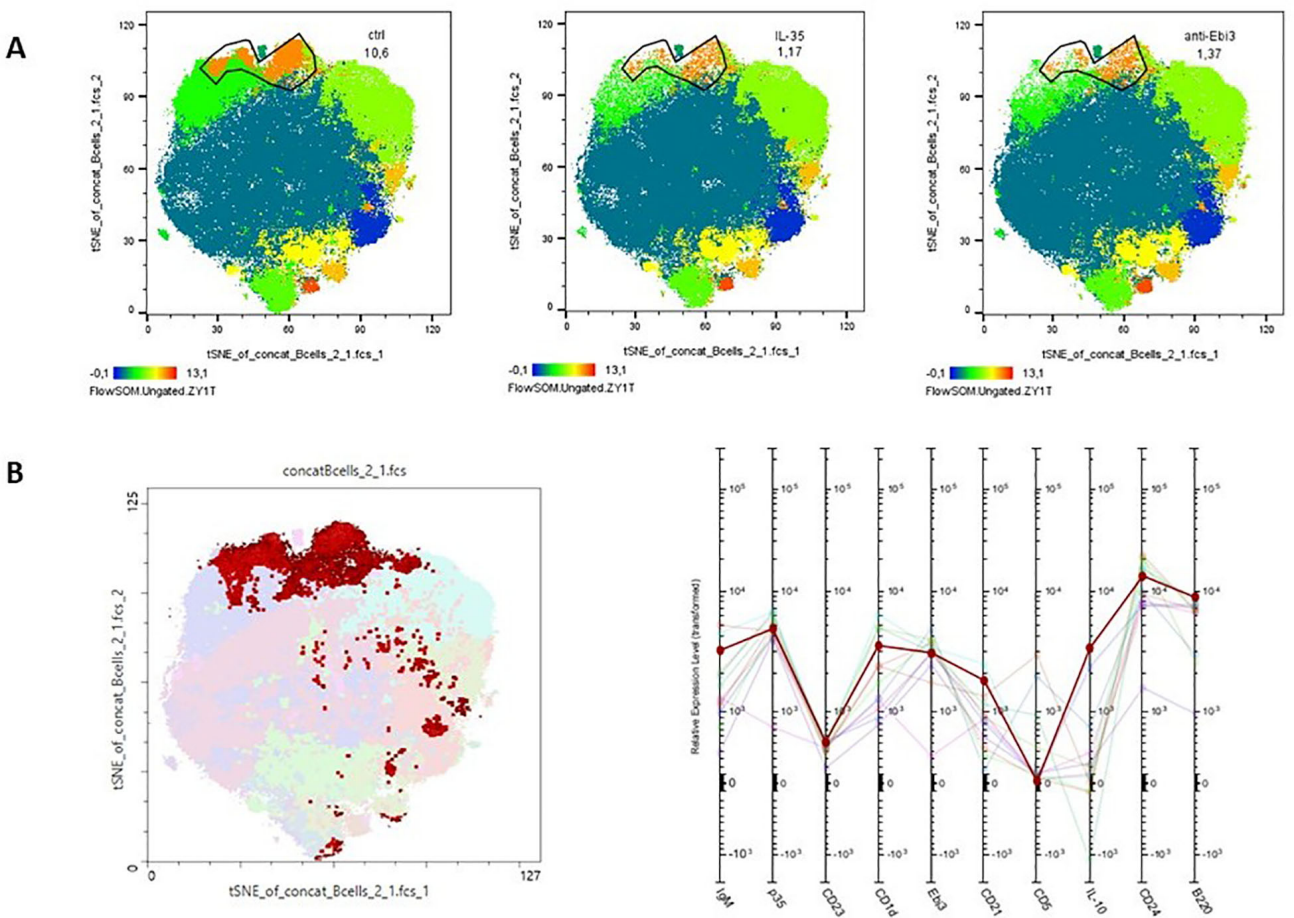
Cluster analysis of B cells from spleen. t-Distributed Stochastic Neighbor Embedding (t-SNE) visualization of clusters identified in the spleen **(A)** following FlowSOM analysis. The profile plots **(B)** represent the relative expression intensity of each parameter across the identified populations. Each line corresponds to a distinct population, with the color of the line matching the legend.

**Figure 6:**
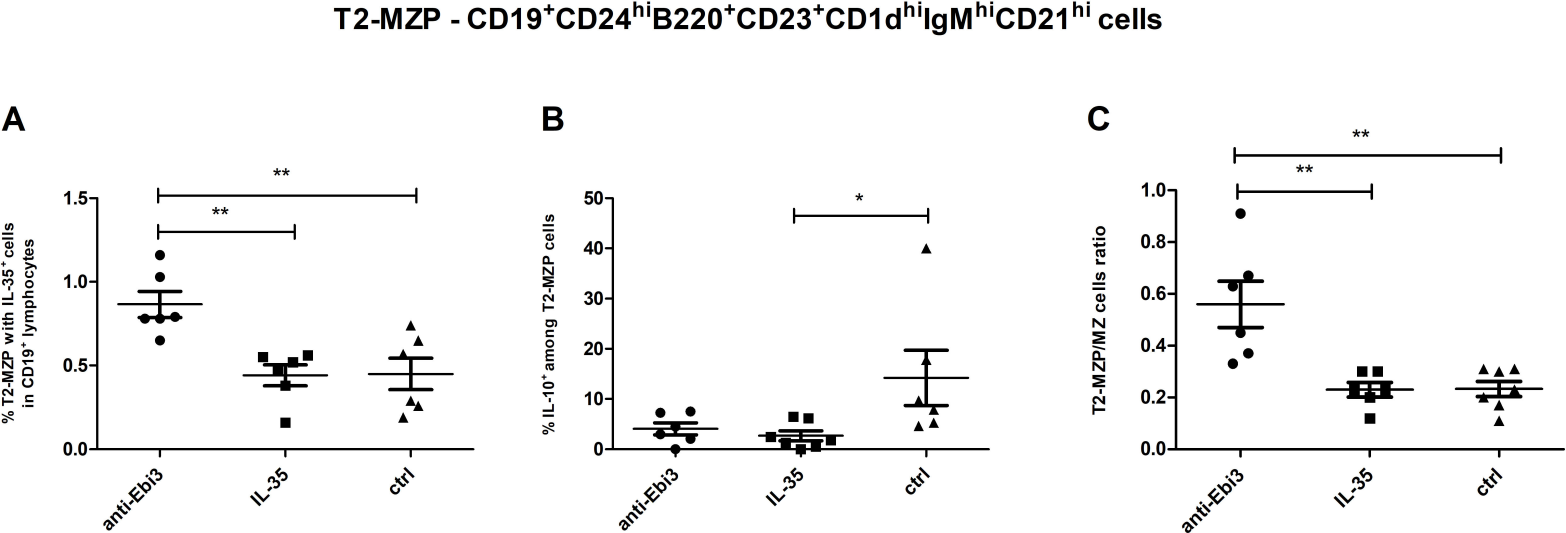
Frequencies of T2-MZP cells **(A)** with IL-35 expression within CD19^+^ lymphocytes, **(B)** with IL-10 expression within T2-MZP cells. T2-MZP/MZ cells ratio **(C)**; n=7. Data are expressed as mean ± SEM. Normality was assessed using the Shapiro–Wilk normality or Kolmogorov–Smirnov tests. *p < 0.05, **p < 0.01 as analyzed by the one-way analysis of variance with Tukey’s multiple comparison test or Krauskal–Wallis test with Dunn’s multiple comparison test.

Advanced analytical methods, including t-SNE and the FlowSOM learning algorithm, facilitate comprehensive cross-sample comparisons using two-dimensional gating strategies. Using FlowSOM, we identified 12 distinct cell subpopulations, each defined by specific expression profiles of selected markers. Comparative analysis of the spleen across the control and experimental groups revealed a cluster (orange cluster; **Figure 5A**) with a reduction in cell numbers in the treated groups (1.17% for IL-35 group and 1.37% for anti-Ebi3 group) relative to that in the controls (10.6%). These findings underscore the significance of cells compatible with the CD19^+^CD24^high^B220^+^CD23^+^CD1d^high^IgM^high^CD21^high^ (regulatory cells - T2-MZP) phenotype based on marker expression (**Figure 5B**), which was subsequently examined using conventional gating methods. Administration of anti-Ebi3/IL-35 antibody increased the T2-MZP^IL-35+^ cell proportion (**Figure 6A**). In turn, rIL-35 administration decreased the number of T2-MZP^IL10+^ cells in comparison to the control group (**Figure 6B**). The number of MZ cells was not different between the examined groups (*p*>0.05). The T2-MZP/MZ cell ratio was also higher in the anti-Ebi3 group (**Figure 6C**). Similarly, the frequency of FO cells was not different (data not shown). Moreover, no cells with the CD19^+^CD24^high^B220^+^CD23^+^CD1d^high^IgM^high^CD21^high^ (T2-MZP) phenotype were detected in the uterine-draining lymph nodes.

### 3.4 IL-35 changes Treg cell frequency at the periphery

**Figure 7:**
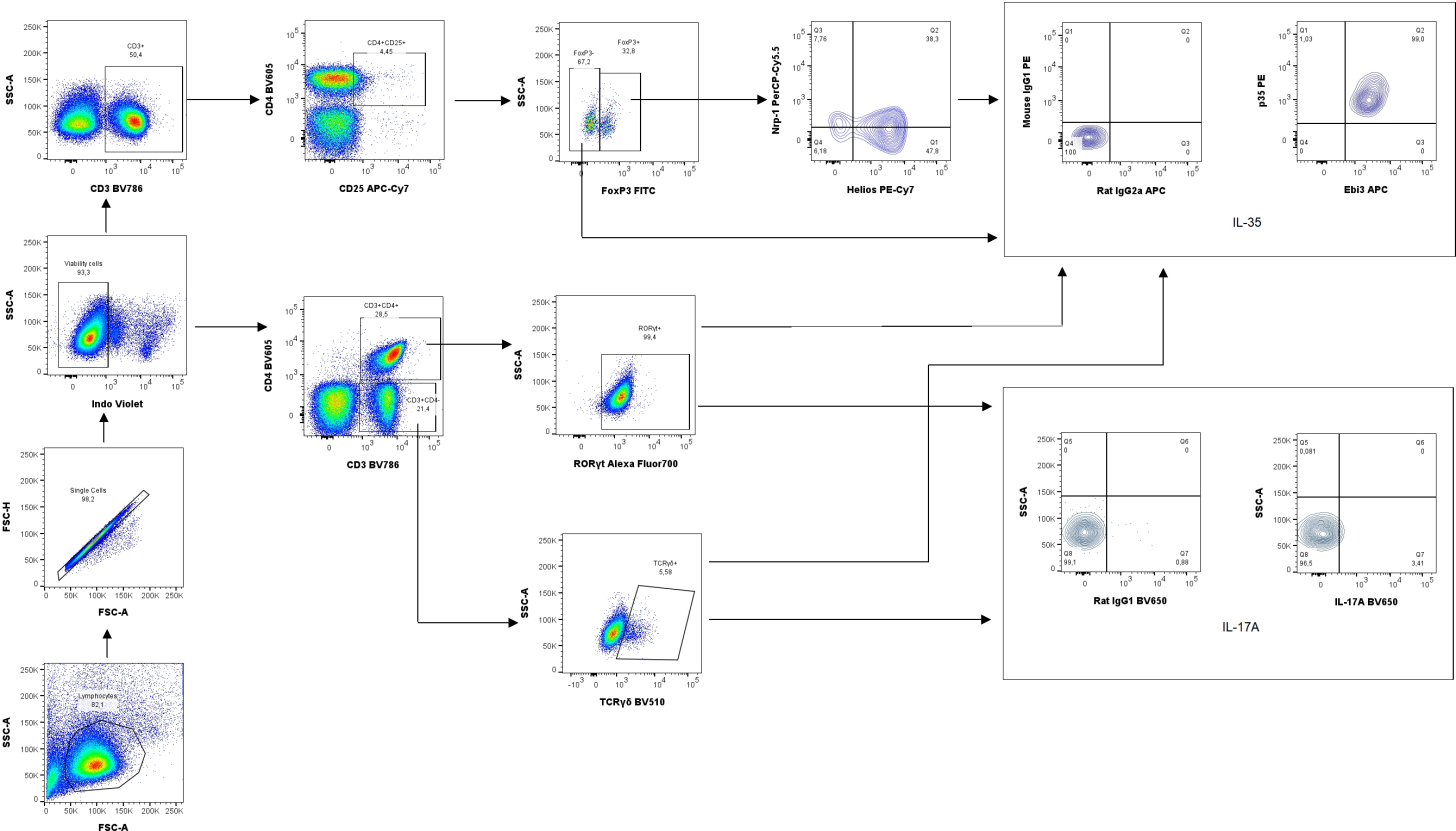
Representative dot-plots showing the gating strategy of regulatory T cells (CD3^+^CD4^+^CD25^+^Foxp3^+^Helios^+/−^Nrp-1^+/−^IL-35^+^), iTr35 cells (CD3^+^CD4^+^CD25^+^Foxp3^−^IL-35^+^), Th17 cells (CD3^+^CD4^+^RORγt^+^IL-17A) and γδ T cells (CD3^+^CD4^+^TCRγδ^+^IL35^+^/IL17A^+^). For cytokine analysis we used appropriate isotype controls in the same concentration as specific antibodies. For other antigens FMO controls were used.

**Figure 8:**
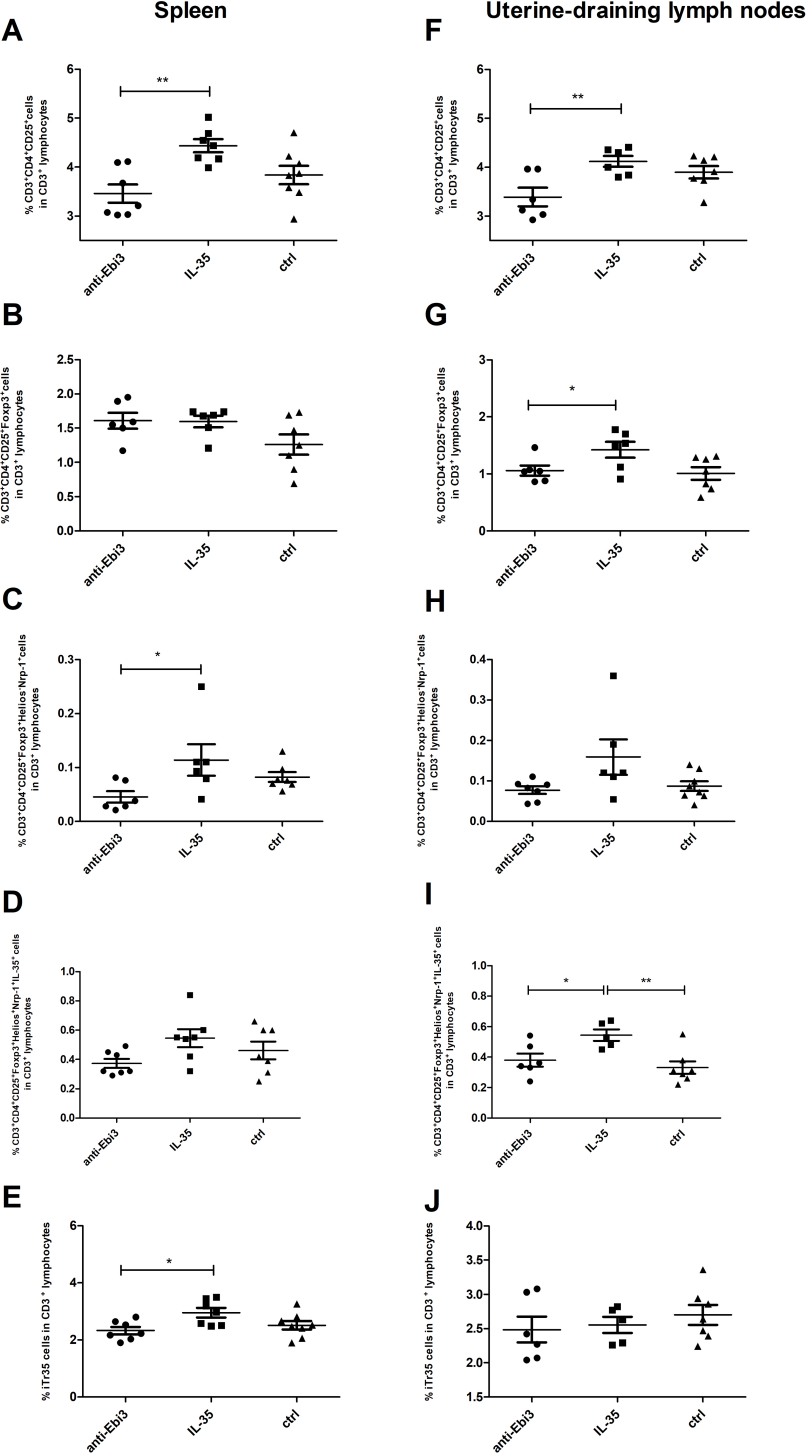
The proportion of activated T cells (CD3^+^CD4^+^CD25^+^), Tregs (CD3^+^CD4^+^CD25^+^Foxp3^+^), Tregs (CD3^+^CD4^+^CD25^+^Foxp3^+^Helios^−^Nrp-1^+^), Tregs (CD3^+^CD4^+^CD25^+^Foxp3^+^Helios^+^Nrp-1^+^IL35^+^), and iTr35 (CD3^+^CD4^+^CD25^+^Foxp3^−^IL-35^+^) cells in the spleen **(A–E)** and uterine-draining lymph nodes **(F–J)**; n=7. Data are expressed as mean ± standard error of the mean (SEM). Normality was assessed using the Shapiro–Wilk normality or Kolmogorov–Smirnov tests. *p < 0.05, **p < 0.01 as analyzed by the one-way analysis of variance with Tukey’s multiple comparison test or Krauskal–Wallis test with Dunn’s multiple comparison test.

Next, the percentage of Treg cells in the spleen and uterine-draining lymph nodes was analyzed based on the gating strategy shown in **Figure 7**. After intraperitoneal IL-35 administration, the percentage of activated T cells (CD3^+^CD4^+^CD25^+^) increased in the spleen (**Figure 8A**) as well as uterine-draining lymph nodes (**Figure 8F**) than that after anti-Ebi3 antibody administration. We also observed alterations in the frequency of Tregs (CD3^+^CD4^+^CD25^+^Foxp3^+^) in uterine-draining lymph nodes (**Figure 8G**), not in spleen (**Figure 8B**). The IL-35 group showed a higher percentage of these cells. We thus examined the phenotypes of Tregs in further detail. Therefore, Helios, Nrp-1, and IL-35 expression levels were assessed. The percentage of CD3^+^CD4^+^CD25^+^Foxp3^+^Helios^−^Nrp-1^+^ cells increased in the spleen, but not in uterine-draining lymph nodes after IL-35 administration than that in the anti-Ebi3 group (respectively **Figures 8C, H**). Moreover, the proportion of CD3^+^CD4^+^CD25^+^Foxp3^+^Helios^+^Nrp-1^+^IL35^+^ cells increased in the uterine-draining lymph nodes, but not in the spleen, after IL-35 administration than that in the anti-Ebi3 and control groups (respectively **Figures 8I, D**). The frequency of iTr35 (CD3^+^CD4^+^CD25^+^Foxp3^−^IL-35^+^) cells increased in the spleen after IL-35 administration (**Figure 8E**). In uterine-draining lymph nodes, the percentage of iTr35 cells did not change between the study groups (**Figure 8J**). We did not observe any changes in frequencies of Th17 cells (CD3^+^CD4^+^RORγt^+^IL-17^+^), and γδ T cells as well as IL-17A production by Th17 and γδ T cells (data not shown).

### 3.5 IL-35 increased NK^IL-35+^ and CD19^+^IL-35^+^ cell frequency and did not change Treg proportion in the decidua

**Figure 9:**
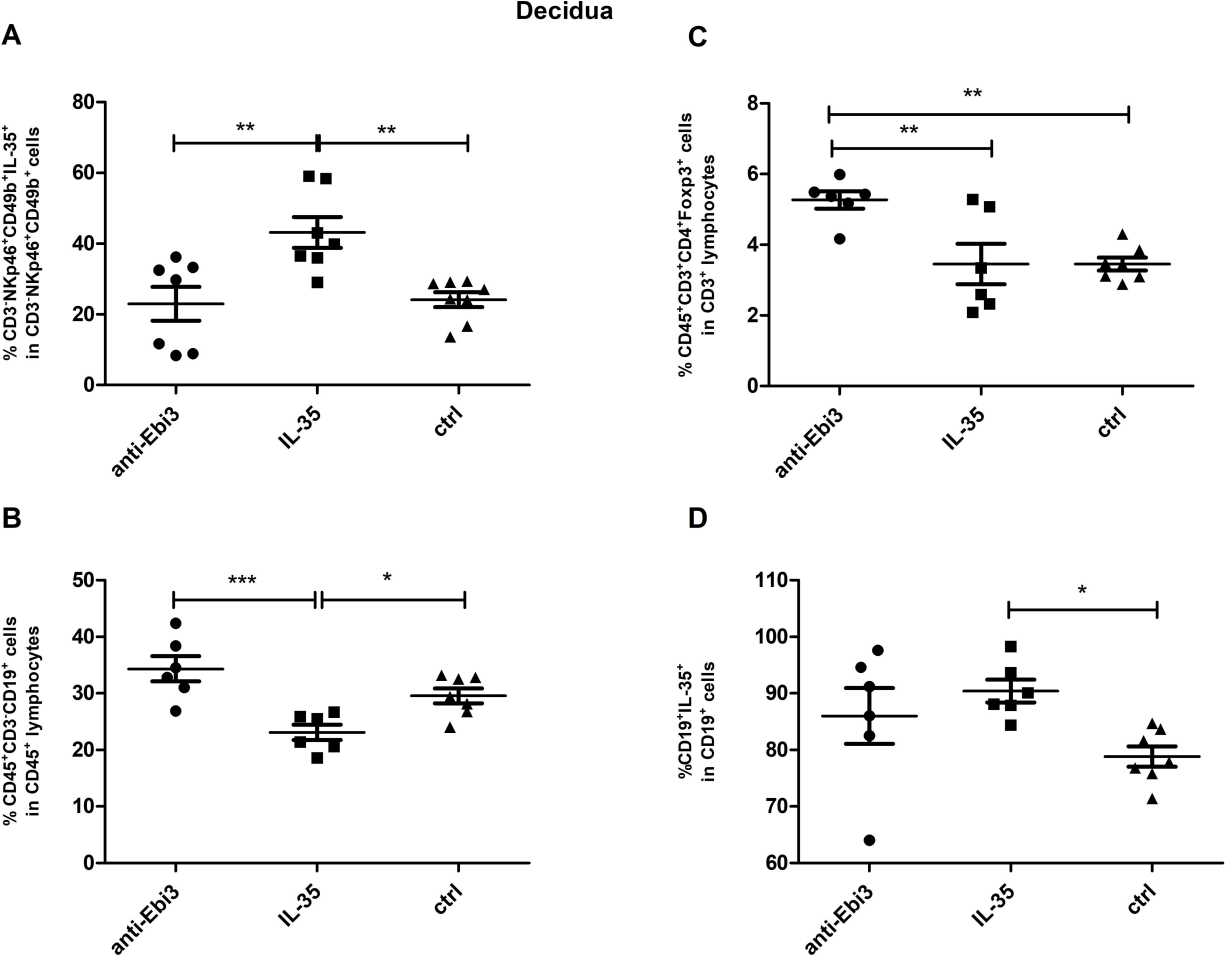
The percentage of: NK (CD3^−^NKp46^+^CD49b^+^IL35^+^) cells **(A)**, regulatory T (CD45^+^CD3^+^CD4^+^Foxp3^+^) cells **(C)**, B (CD45^+^CD3^+^CD19^+^) cells **(B)** and B cells producing IL-35 **(D)** in decidua of DBA/2J- mated CBA/J female at 14 days *post coitum* (dpc); n=7. Data are expressed as mean ± standard error of the mean (SEM). Normality was assessed using the Shapiro–Wilk normality or Kolmogorov–Smirnov tests. *p < 0.05, **p < 0.01, ***p < 0.001 as analyzed by the one-way analysis of variance with Tukey’s multiple comparison test or Krauskal–Wallis test with Dunn’s multiple comparison test.

We observed that the proportion of IL-35-producing NK cells (CD3^−^NKp46^+^CD49b^+^IL35^+^) increased in the decidua after IL-35 application (**Figure 9A**). The frequency of CD19^+^ lymphocytes decreased in the IL-35 group than in the control and anti-Ebi3 groups (**Figure 9B**). Interestingly, CD19^+^IL35^+^ cells increased after IL-35 administration than in the control group (**Figure 9D**). Blocking Ebi3 affected the increased proportion of Tregs than the other two treatments (**Figure 9C**). However, the number of iTr35 (CD45^+^CD3^+^CD4^+^Foxp3^−^Ebi-3^+^p35^+^) cells were not different between the examined groups (data not shown).

### 3.6 IL-35 and anti-Ebi3 do not affect NK cell maturation

**Figure 10:**
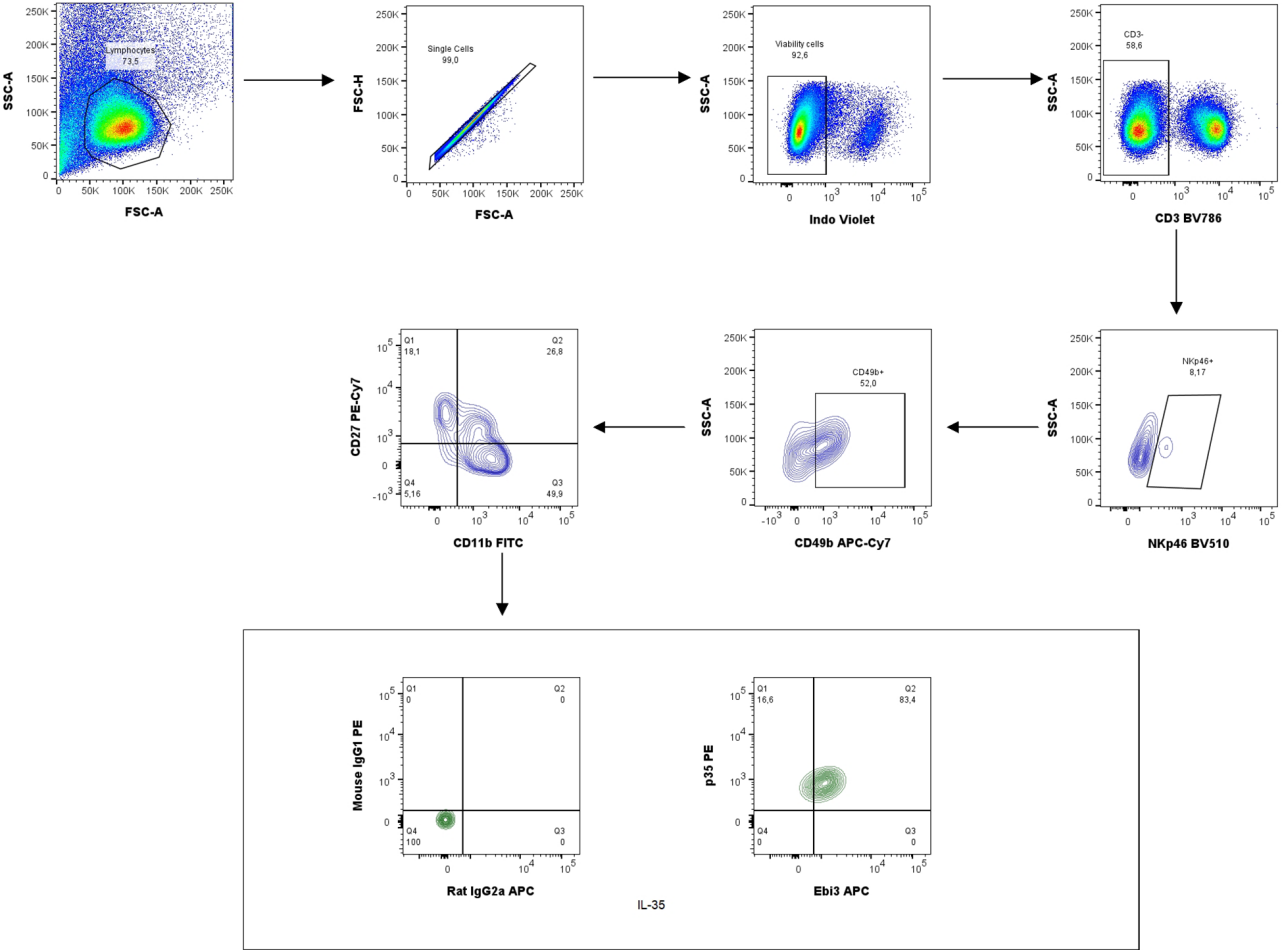
Representative dot-plots showing the gating strategy of NK cells: immature NK cells (CD3^−^NKp46^+^CD49b^+^CD27^+^CD11b^−^), M1 NK cells (CD3^−^NKp46^+^CD49b^+^CD27^+^CD11b^+^) and M2 NK cells (CD3^−^NKp46^+^CD49b^+^CD27^−^CD11b^+^). For cytokine analysis we used appropriate isotype controls in the same concentration as specific antibodies. For other antigens FMO controls were used.

**Figure 11:**
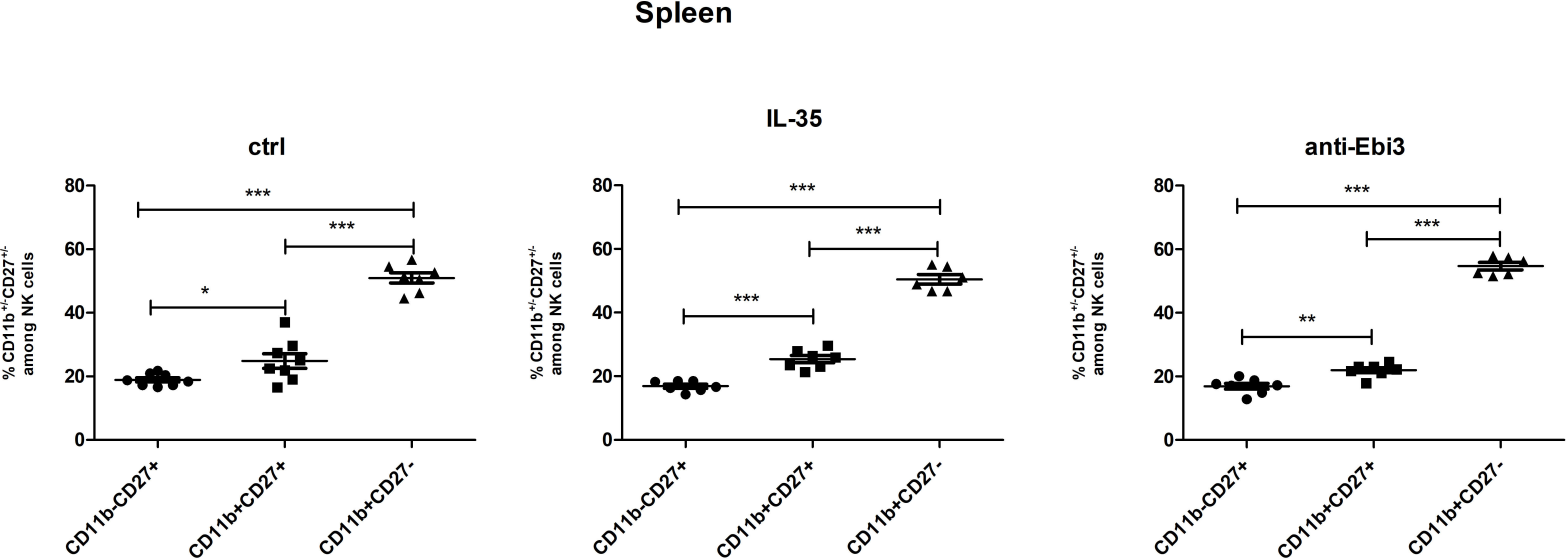
The frequencies of immature (CD3^−^NKp46^+^CD49b^+^CD27^+^CD11b^−^), M1 (CD3^−^NKp46^+^CD49b^+^CD27^+^CD11b^+^), and M2 (CD3^−^NKp46^+^CD49b^+^CD27^−^CD11b^+^) NK cells in all examined groups (ctrl, IL-35 and anti-Ebi3); n=7. Data are expressed as mean ± standard error of the mean (SEM). Normality was assessed using the Shapiro–Wilk normality or Kolmogorov–Smirnov tests. *p < 0.05, **p < 0.01, ***p < 0.001 as analyzed by the one-way analysis of variance with Tukey’s multiple comparison test.

Next, the percentage of NK cells in the spleen was analyzed based on the gating strategy shown in **Figure 10**. IL-35 and anti-Ebi3 antibody administration do not affect NK cell maturation. We did not observe any differences in the proportion of the tested subpopulations between the examined groups. The frequencies of the three tested subpopulations: immature NK cells (CD3^-^NKp46^+^CD49b^+^CD27^+^CD11b^-^), M1 NK cells (CD3^-^NKp46^+^CD49b^+^CD27^+^CD11b^+^), and M2 NK cells (CD3^-^NKp46^+^CD49b^+^CD27^-^CD11b^+^) were similar in all examined groups (**Figure 11**).

### 3.7 The effect of IL-35 on serum cytokine levels

**Figure 12:**
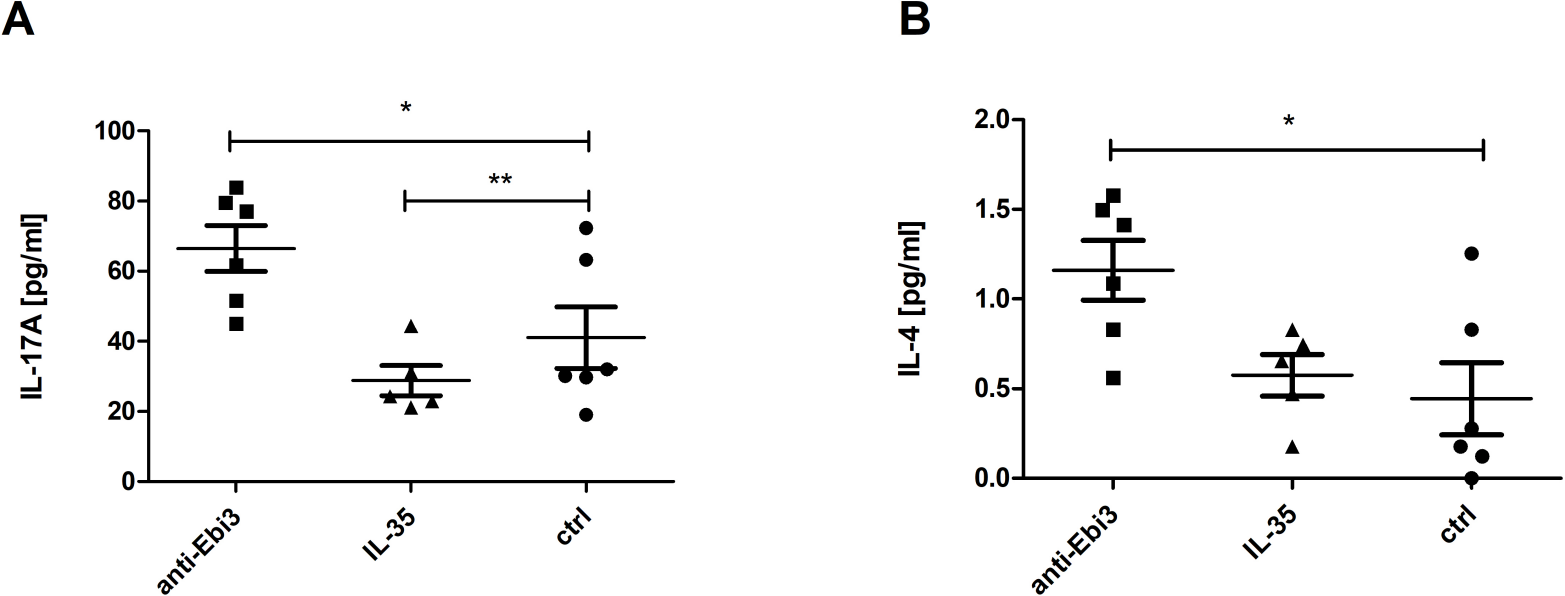
Interleukin (IL)-17A **(A)** and IL-4 **(B)** levels in mouse serum. Data are expressed as mean ± standard error of the mean (SEM). Normality was assessed using the Shapiro–Wilk normality or Kolmogorov–Smirnov tests. *p<0.05, **p<0.01, as analyzed by the one-way analysis of variance with Tukey’s multiple comparison test.

Out of the cytokines studied (GM-CSF, IFN-γ, IL-1β, IL-2, IL-4, IL-5, IL-6, IL-9, IL-10, IL-12p70, IL-13, IL-17A (CTLA-8), IL-18, IL-22, IL-23, IL-27, IL-33, TNF-α, IL-35, TGF-β), we observed changes only in IL-17A and IL-4. After anti-Ebi3 administration, IL-17A levels increased in the mouse serum (**Figure 12A**). IL-35 delivery blocked the increase in IL-17A levels. IL-4 levels increased after anti-Ebi3 administration (**Figure 12B**). The IL-35 levels in all tested samples were below the lowest standard curve point (0.47 ng/mL). Also, cytokine (IL-9, IL-13, IL-18, IL-33, IFN-γ) determinations were not possible due to the lack of minimum levels in many of the tested samples. Graphs showing the levels of remaining cytokines are provided in the **Supplementary Data**.

## 4 Discussion

Determination of appropriate immunological interactions between the mother and fetus and the establishment of immune tolerance are fundamental prerequisites for a successful pregnancy. The contribution of immunoregulatory T and B lymphocytes in normal pregnancy development as well as the cytokine microenvironment remains unclear. Considering the unique features of IL-35, such as induction of infection tolerance, it seems to be crucial for successful pregnancy. Therefore, this study aimed to investigate the influence of intraperitoneal rIL-35 and anti-Ebi3 antibody administration within a short time after mating on successful pregnancy, fetal blood flow, and the profile of several types of regulatory cells in murine AP model (CBA/J♀ x DBA/2J♂).

In our study, we did not observe any differences in abortion rates between the examined groups. However, Liu et al. (15) demonstrated a positive effect of IL-35 administration on abortion rate in the same animal model. Neutralizing IL-35 antibody was also administered in their research. The results displayed that treatment with anti-IL-35 mAb enhanced abortion rates and decreased IL-35 expression in naive conventional T cells (Tconv). Notably, both experiments were different. Apart from the difference in the size of examined groups and statistical analysis methods used, Liu et al. administered 0.75 µg rIL-35 per day from day 2 to day 12 of pregnancy. Moreover, based on many premises presented below, we decided to use a single 2 µg IL-35 dose shortly after mating, at day 0 of pregnancy. This was the original intent of our project, for which we applied for funding before Liu’s work was published. The adoptive transfer of B10 cells from NP females to AP animals, which decreased the number of resorbed fetuses, increased the number of Treg cells (CD4^+^CD25^+^Foxp3^+^) in the lymph nodes, and reduced CD80 expression on the dendritic cell surface (DC tolerogenic phenotype), was performed on day 0 of pregnancy (30). Moreover, we have previously shown significantly lower levels of IL-35-producing Breg cells on day 3 of pregnancy in AP mice than in NP mice, indicating early impairment of pregnancy immune tolerance. Similarly, adoptive transfer of pregnancy-induced CD4^+^CD25^+^ regulatory T cells reversed the IL-17-mediated increase in abortion rate in the CBA/JxBALB/c mouse model when performed 2 days before mating. Treg transfusion on day 7 of pregnancy (after implantation) had no effect (44). Adoptive Treg transfer from NP mice to AP mice was also performed before implantation, at 0–2 day of gestation (45). Therefore, studies by Ann Zenclussen’s group indicate the success of any intervention (such as adoptive transfer of B10 or Treg cells) if they occur shortly after mating, before implantation. Moreover, a one-time supply eliminated the impact of stress on pregnancy success. Our many years of experience with this mouse strain indicate that stress significantly impacts pregnancy success. We found that this single early dose was not sufficient to prevent pregnancy loss. Although IL-35 administration increased the frequency of B10 cells, IL-35-producing B10 cells, Tregs and IL-35-producing Tregs at the periphery, as well as CD19^+^IL-35^+^ B cells in decidua, decidual Tregs remain unchanged. The lack of Treg expansion at the fetal–maternal interface is likely the main reason why single-dose IL-35 treatment failed to confer protection in our model. Moreover, multiple studies demonstrate the indispensable role of decidual Tregs in pregnancy maintenance (46). Importantly, in human decidua, Bregs and Tregs are found in close proximity, forming clusters that support the possibility of functional interactions. IL-35 may act bidirectionally in this context, facilitating both Breg and Treg proliferation (47). Well-timed interaction of correctly functioning maternal immune cells is essential for pregnancy success. Therefore, we can surmise that a single early signal of IL-35 was too weak to expand a sufficiently effective decidual Treg pool to prevent abortion, whereas repeated IL-35 exposure later in gestation, as in Liu et al., provides a stronger and sustained signal capable of protecting pregnancy.

Although we did not observe a protective effect against miscarriage, administering a single rIL-35 and anti-Ebi3 neutralizing antibody dose induced numerous effects on regulatory B and T lymphocytes at the local and peripheral levels. IL-35 administration increases the frequency of B10 cells in uterine-draining lymph nodes. Moreover, the frequency of these cells with IL-35 expression was also higher in the uterine-draining lymph nodes as well as spleen after IL-35 administration. Interestingly, the frequency of B10^IL-10+^ and IL-35-producing B10 (B10^IL-35+^) cells in the spleen differed. After IL-35 and anti-Ebi3 antibody administration, we observed a decrease in the frequency of B10^IL-10+^ cells. Wei et al. (48) demonstrated that IL-35 and IL-10 expression define two distinct Treg subsets with different activation statuses and transcription factor dependencies. Blimp1 is critical for IL-10 production, but not IL-35 production, whereas Foxp3 is essential for IL-35 production but dispensable for IL-10 production. It is possible that the B10 cells can also be divided into two subpopulations: IL-10-producing (B10^IL-10+^) and IL-35-producing (B10^IL-35+^) lymphocytes. IL-35 induces Bregs and promotes their conversion into a Breg cell subset that produces IL-35 and IL-10. Adoptive Breg cell transfer (induced by recombinant IL-35) to mice with established disease suppresses EAU by inhibiting T helper type 17 (Th17) and Th1 responses and promoting Treg cell expansion (21). Here, we observed that IL-35 induces B10^IL-35+^ cell expansion. Anti-Ebi3 antibody administration did not influence the examined cell populations in the uterine lymph nodes compared to those in the control. However, similar to IL-35, it decreased B10^IL-10+^ cell frequency and influenced T2-MZP lymphocyte frequency in the spleen.

Advanced analytical methods, including t-SNE and FlowSOM learning algorithm, underscored the significance of cells exhibiting the CD19^+^CD24^high^B220^+^CD23^+^CD1d^high^IgM^high^CD21^high^ (T2-MZP) phenotype, which was subsequently examined using conventional gating methods. Evans et al. (49) showed that T2-MZP B cell transfer can prevent the development of arthritis in a DBA/1 collagen-induced arthritis (CIA) model of rheumatoid arthritis (RA). The immunoregulatory function of T2-MZP B cells depends on IL-10 production. Moreover, they inhibit the pathogenic Th1 response *in vivo* and delayed-type hypersensitivity (DTH) response during CIA induction phase (49). T2-MZP B cells suppress IFN-γ production by releasing IL-10 (49). In addition, T2-MZP B cells also significantly reduce CD4^+^IFN-γ^+^ and IL-17^+^ T cell proportion and contribute to differentiation into CD4^+^Foxp3^+^ T cells when cultured with effector CD4^+^CD25^−^ T cells (50, 51). The authors also showed that only T2-MZP Bregs, and not marginal zone (MZ) or follicular (FO) B cell subsets, increased Treg frequency after adoptive transfer to B cell-deficient mice (50). On the contrary, T2-MZP cells accumulated in tumor-draining lymph nodes and promoted tumor (B16-F10 melanoma) growth, but did not increase IL-10 levels; therefore, IL-10 is likely not an exclusive regulatory mechanism utilized by this subset (52). In this study, we observed a decrease in the proportion of IL-10-expressing T2-MZP cells within T2-MZP cells after IL-35 administration. In turn, anti-Ebi3 antibody administration increased the number of T2-MZP^IL-35+^ cells and the T2-MZP/MZ cell ratio. Moreover, we did not observe any changes in MZ or FO cell frequencies among the examined groups. Other studies have indicated that MZ B cell numbers and the MZ/FO B cell ratio increased in the spleens of NP mice, but not in those suffering from pregnancy disturbances (53). IL-35 and anti-Ebi3 administration did not affect the frequency of MZ B cells (data not shown). Thus, the presence of IL-35 in the microenvironment may affect T2-MZP cells and determine which cytokines they produce, IL-35 or IL-10.

We also focused on Tregs that produce IL-35. We observed an increase in the proportion of Treg after IL-35 administration in the spleen and uterine-draining lymph nodes. These populations differed in the expression of transcription factor Helios. Helios and Nrp-1 were initially used for differentiate thymic Tregs (tTregs) from peripheral Tregs (pTregs), but the differences in their expression level did not distinguish thymus-derived from extrathymically-induced CD4^+^Foxp3^+^ Treg cells (54). The Helios^+^ and Helios^−^ Treg subpopulations are phenotypically and functionally distinct and express dissimilar TCR repertoires; moreover, Foxp3 expression is stable in Helios^+^Tregs (55). The number of CD3^+^CD4^+^CD25^+^Foxp3^+^Helios^+^Nrp-1^+^IL35^+^ population increased in the uterine-draining lymph nodes after IL-35 administration compared to the control group. Therefore, IL-35 may have a stabilizing effect on Foxp3 expression in Helios^+^Tregs. The novel subpopulation of Treg cells (iTr35), characterized by phenotypic stability and potent immunosuppressive function through IL-35 production and the lack of Foxp3 expression, also seems to be crucial for pregnancy success (5). We observed an increased frequency of iTr35 cells in the spleen after IL-35 administration than that after anti-Ebi3 injection. This is a subtle change that we do not see compared to the control group. The control group had baseline IL-35 levels, and only blocking IL-35 affected the observed difference. However, prolonged IL-35 administration by Liu et al. 15 increased the frequency of decidual CD4^+^ cells expressing both Ebi3 protein and mRNA. These cells are referred to as iTr35 cells. In this study, we investigated IL-35 expression in a population of CD3^+^CD4^+^CD25^+^Foxp3^−^Ebi3^+^p35^+^ lymphocytes. Precise immunophenotyping of iTr35 cells is a probable reason for the discrepancy observed between our results and those of Liu et al.

Our study showed that IL-35 administration influenced the frequency of decidual B and NK cells in contrary to anti-IL-35 deposition, which in turn influenced the Treg cell frequency. The CD19^+^ cell frequency decreased in the IL-35 group than in the control and anti-Ebi3 groups. Interestingly, the percentage of CD19^+^IL35^+^ cells increased after IL-35 administration compared to the control group. Previously, we noted that the CD19^+^IL-35^+^ percentage in the uterus (3 dpc) and decidua (14 dpc) was lower in an AP model compared to a normal pregnancy (NP model) (6). In contrast, the number of iTr35 (CD45^+^CD3^+^CD4^+^Foxp3^-^Ebi-3^+^p35^+^) cells was not different between the examined groups (data not shown); however, the CD45^+^CD3^+^CD4^+^Foxp3^+^ cell frequency significantly increased after anti-Ebi3 administration. We wondered whether this could be a compensatory mechanism, in which blocking IL-35 increases the number of Tregs. The proportion of IL-35-producing NK cells (CD3^−^NKp46^+^CD49b^+^IL35^+^) increased in the decidua after IL-35 application. During pregnancy, NK cells from the uterus and maternal–fetal interface are involved in placental vascular remodeling, regulating invading trophoblast cells, and providing immunity (56); therefore, our observations seem to be important for successful pregnancy. Additionally, we determined if anti-Ebi3 and IL-35 administration affected NK cell maturation. CD11b and CD27 markers were used to divide NK cells into three maturation subsets: immature (CD3^−^NKp46^+^CD49b^+^CD27^+^CD11b^−^), mature 1 (M1, CD3^−^NKp46^+^CD49b^+^CD27^+^CD11b^+^), and mature 2 (M2, CD3^−^NKp46^+^CD49b^+^CD27^−^CD11b^+^) (57, 58). These subsets differed in their proliferative and cytotoxic capacities. More mature cells become more cytotoxic and lose their proliferative potential (58, 59). In our study, the frequencies of the three tested subpopulations in the spleen were similar in all examined groups which indicates no effect of IL-35 on NK cell maturation.

IL-35, as a strong pleiotropic cytokine, also exerts a systemic influence. IL-35 targets not only conventional/effector T cells, Treg cells, and Breg cells, but also non-immune cells, including aortic endothelial cells (60). Therefore, we examined the cytokine profile in blood sera and umbilical blood flow in 14-day fetuses. We observed a significantly lower embryonic HR and less-developed umbilical vessels after anti-Ebi3 administration. The PI, a noninvasive and easy method for obtaining parameters with a broad range of research and clinical applications, was higher in this group than in the control group. It is generally believed that the PI of Doppler recordings in the umbilical arteries is a marker of the circulatory competence of the fetal placenta (61). In human pregnancy, high PI values of the umbilical artery correlate with fetal growth restriction markers and an increased risk of moderate and severe small-for-gestational-age at birth (62). In mid-gestation (10.5 dpc), an increased PI and resistance of the uterine arteries were observed in a mouse model of Treg deficiency, suggesting profound vessel impairment. Moreover, Treg depletion reduced Ebi3, p35, IL-10, and TGFβ mRNA expression. In contrast, IFNγ mRNA expression remains unchanged, whereas Vegfa expression was reduced than in the control mice. Ncr1, which encodes the uNK cell maturation marker NKp46, was also reduced in this study, which is consistent with the histological finding of fewer uNK cells in decidual tissues. The authors concluded that Treg cells (and particularly IL-35, a cytokine unique to Tregs) influenced NK cell frequency and possibly influenced placental vessel development (63). Our results confirmed that the restriction of IL-35 availability after anti-Ebi3 antibody administration may affect placental impedance to blood flow. However, compared to control mice, we observed that the frequency of decidual Tregs (CD45^+^CD3^+^CD4^+^Foxp3^+^), but not Tregs, expressing IL-35 (CD45^+^CD3^+^CD4^+^Foxp3^+^IL-35^+^) (data not shown, no difference between examined groups) increased after delivering this antibody. It can also be assumed that the antibody used neutralizes IL-35 originating from other sources, such as the trophoblast itself, or eliminates its protective effects within the vessels (16, 64). In addition, the frequency of IL-35-producing decidual NK and B cells increased after rIL-35 administration. These results may indirectly indicate the cooperation of these cells and the positive effect of IL-35 on the circulatory competence of the fetal placenta.

Elevated Th17/Th2 (IL-17A and IL-4) cytokine concentrations are observed at the periphery in anti-Ebi3 group compared to the control group. On the one hand, we observed an increase in IL-17A levels. Th17 cells adversely affect pregnancy success through the impairment of regulatory mechanisms. Liu et al. (65) showed that the proportion of Th17 cells and IL-17A concentrations were significantly higher in patients with unexplained recurrent spontaneous abortion (URSA) than in patients with normal early pregnancy and non-pregnant patients. Moreover, the Th17 to Treg ratio was significantly higher in the URSA group than in the other two groups. Some evidence suggests that the development of Th17 and induced Treg cells during the immune response are mutually exclusive (66) and that the Th17/Treg system is similar to the Th1/Th2 balance. Therefore, the role of IL-35 as a cytokine regulating the Th17/Treg balance during pregnancy seems to be notable. On the other hand, Th17 cells are not always harmful during pregnancy. The Th17-type cytokines IL-17 and IL-22 together could have both positive and negative impacts on pregnancy. This depends on the cytokine milieu and can differentiate into Th17/Th1 or Th17/Th2 cells. Th17/Th2 cells (producing IL-17 or IL-22 and IL-4) have been found in the decidua of successful pregnancies, and support embryo implantation and pregnancy (67, 68). This may be another compensatory mechanism triggered by reduced or absent IL-35 availability.

In summary, a single administration of IL-35 shortly after mating was not sufficient to prevent pregnancy loss. The lack of a protective effect against miscarriage may result from an insufficiently strong stimulatory signal to promote tolerance. Although IL-35 administration increased the frequency of B10 cells, IL-35-producing B10 cells, Tregs and IL-35-producing Tregs at the periphery, as well as CD19^+^IL-35^+^ B cells in decidua, decidual Tregs remain unchanged. The lack of Treg expansion at the fetal–maternal interface is likely the main reason why single-dose IL-35 treatment failed to confer protection in our model. Moreover, IL-35 neutralization in AP mice showed the systemic significance of the availability of this cytokine in maintaining the cytokine balance, which manifested as an increased Th17/Th2 response in the periphery. Additionally, the restriction of IL-35 availability resulted in decreased embryonic heart rate and impaired placental–fetal circulation. Our results may indicate a potential involvement of IL-35 in the establishment of tolerance during pregnancy through its multifaceted effects on regulatory cells. However, its complex pleiotropic effects on different populations of regulatory cells require further investigation.

## Data Availability

The raw data supporting the conclusions of this article will be made available by the authors, without undue reservation.

## Supplementary material

Supplementary Figure S1 The levels of interleukins: GM-CSF, IL-1beta, IL-2, IL-5, IL-6, IL-10, IL-12p70, IL-22, IL-23, IL-27, TNF-alpha, IL-35, TGF-beta in mouse serum. Data are expressed as mean ± standard error of the mean (SEM). Normality was assessed using the Shapiro–Wilk normality or Kolmogorov–Smirnov tests. No differences between examined groups as analyzed by the one-way analysis of variance or Kruskal–Wallis test.
