# Supplementary figures and images for: The recombinant IL-35 and anti-Ebi3 antibody administration before implantation modulate immune regulation and fetal outcomes in an abortion-prone mouse model
Source: Front Immunol. 2025 Nov 19;16:1648641. doi: 10.3389/fimmu.2025.1648641 (PMC12672315; doi:10.3389/fimmu.2025.1648641)

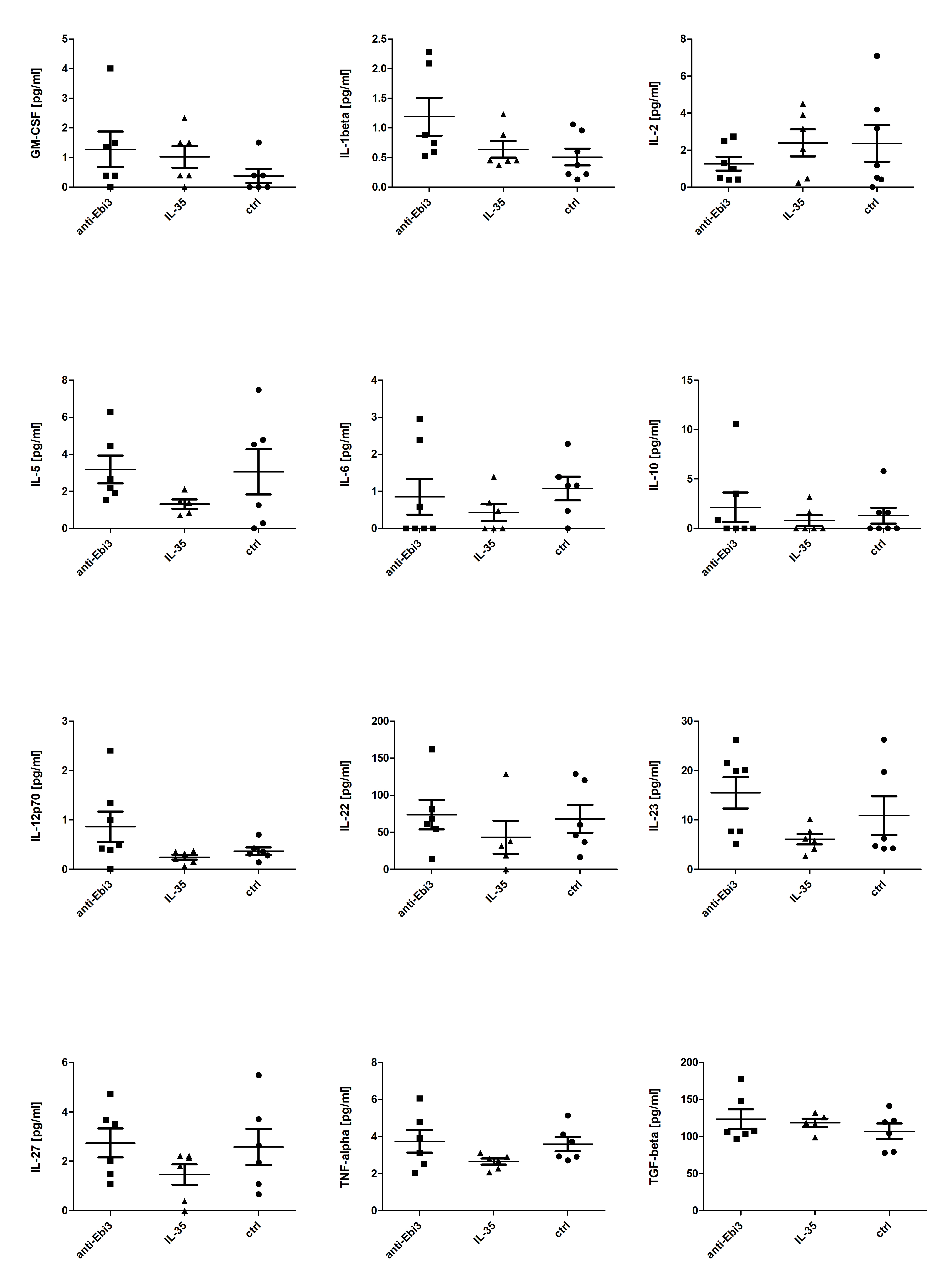

Supplement: Supplementary Figure S1 — The levels of interleukins: GM-CSF, IL-1beta, IL-2, IL-5, IL-6, IL-10, IL-12p70, IL-22, IL-23, IL-27, TNF-alpha, IL-35, TGF-beta in mouse serum. Data are expressed as mean ± standard error of the mean (SEM). Normality was assessed using the Shapiro–Wilk normality or Kolmogorov–Smirnov tests. No differences between examined groups as analyzed by the one-way analysis of variance or Kruskal–Wallis test. [file Image1.jpeg]
